# It takes two—coincidence coding within the dual olfactory pathway of the honeybee

**DOI:** 10.3389/fphys.2015.00208

**Published:** 2015-07-28

**Authors:** Martin F. Brill, Anneke Meyer, Wolfgang Rössler

**Affiliations:** Behavioral Physiology and Sociobiology, Biozentrum, University of WürzburgWürzburg, Germany

**Keywords:** olfaction, insect, coincidence, multi-electrode-recording, antennal lobe, mushroom body

## Abstract

To rapidly process biologically relevant stimuli, sensory systems have developed a broad variety of coding mechanisms like parallel processing and coincidence detection. Parallel processing (e.g., in the visual system), increases both computational capacity and processing speed by simultaneously coding different aspects of the same stimulus. Coincidence detection is an efficient way to integrate information from different sources. Coincidence has been shown to promote associative learning and memory or stimulus feature detection (e.g., in auditory delay lines). Within the dual olfactory pathway of the honeybee both of these mechanisms might be implemented by uniglomerular projection neurons (PNs) that transfer information from the primary olfactory centers, the antennal lobe (AL), to a multimodal integration center, the mushroom body (MB). PNs from anatomically distinct tracts respond to the same stimulus space, but have different physiological properties, characteristics that are prerequisites for parallel processing of different stimulus aspects. However, the PN pathways also display mirror-imaged like anatomical trajectories that resemble neuronal coincidence detectors as known from auditory delay lines. To investigate temporal processing of olfactory information, we recorded PN odor responses simultaneously from both tracts and measured coincident activity of PNs within and between tracts. Our results show that coincidence levels are different within each of the two tracts. Coincidence also occurs between tracts, but to a minor extent compared to coincidence within tracts. Taken together our findings support the relevance of spike timing in coding of olfactory information (temporal code).

## Introduction

Animals process sensory input rapidly in order to behave adequately in their natural environment. In order to manage this challenging task, neural systems have developed a broad variety of mechanisms. Among these, parallel processing and coincidence detection appear to be almost universally useful throughout modalities and animal taxa. Parallel processing codes different aspects of the same stimulus along separate pathways. This way it increases both computational capacity and overall processing speed (Nassi and Callaway, 2009). In contrast, coincidence detection is an efficient way to integrate information from different sources and form association between these, to eventually promote learning (Hebb, 1949; Bliss and Lømo, 1973; Heisenberg, 2003) or stimulus feature detection (Jeffress, 1948; Hassenstein and Reichardt, 1951). In the honeybee olfactory system either one of these mechanisms could potentially be realized by projection neurons (PNs) that transfer information from the primary olfactory neuropile, the antennal lobe (AL) to the multimodal integration center, the mushroom body (MB).

Parallel processing is most prominently known from the vertebrate visual system (Livingstone and Hubel, 1988), where color and shape of a stimulus are analyzed in parallel with a possible motion of the stimulus. Similar distribution of stimulus features on different pathways has been described in the auditory (Rauschecker and Scott, 2009) and the somatosensory systems (Gasser and Erlanger, 1929; Reed et al., 2005). In insects, parallel pathways were described both in vision (Ribi and Scheel, 1981; Fischbach and Dittrich, 1989; Strausfeld et al., 2006; Paulk et al., 2008, 2009) and audition (Helversen and Helversen, 1995). More recently, advances have been made to investigate the role of parallel processing in vertebrate olfaction. These works indicate a division of olfactory bulb output into parallel channels of olfactory information mediated by mitral and tufted cells (Fukunaga et al., 2012; Igarashi et al., 2012; Payton et al., 2012). The two output tract responses differ in their phase to the respiratory oscillation cycle and in detail, tufted cell phase is unperturbed in response to purely excitatory odorants, whereas mitral cell phase is advanced in a graded, stimulus-dependent manner (Fukunaga et al., 2012). However, the existence of a similar spike timing mechanism in insects remains uncertain (Galizia and Rössler, 2010; Sandoz, 2011).

**Figure 1:**
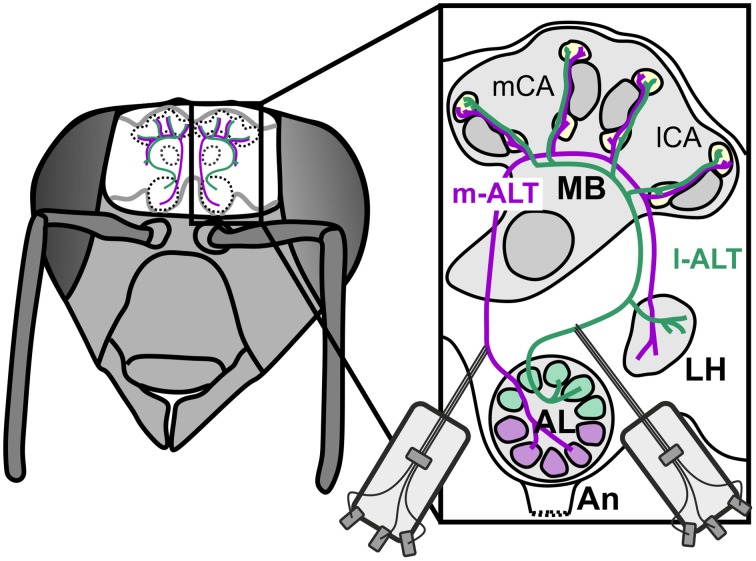
**Schematic overview of the dual olfactory pathway and its connectivity with upstream neurons into the mushroom body of the honeybee brain**. Drawing of a bee's head with the brain exposed and arrangement of the dual olfactory pathway in both brain hemispheres (purple: m-ALT, green l-ALT). Magnification of one hemisphere illustrating the innervation pattern of the m-ALT (purple) and l-ALT (green). Uniglomerular PNs of the dorsal AL hemilobe first innervate the MB lip and basal ring (Br) and finalize their innervation in the lateral horn (LH), giving rise to the m-ALT. The l-ALT emerges from ventral AL glomeruli runs to the LH and later innervates the olfactory MB input sites. An, Antennal nerve; AL, Antennal Lobe; vL, vertical Lobe; mCa and lCa, medial and lateral Calyx; Co, collar.

In favor of potential roles of parallel processing and spike timing, recent anatomical work in the honeybee (Kirschner et al., 2006) and other Hymenoptera (Rössler and Zube, 2011) has shown a dual tract system that pervades from the sensory input stage at the antenna to higher level processing in the MB (Figure 1). Olfactory receptor neurons (ORNs) provide mainly redundant input (Carcaud et al., 2012; Galizia et al., 2012) to the two prominent subsystems of the AL: the ventral and dorsal hemilobe (Kirschner et al., 2006). The ventral hemilobe comprises about 88 spheroidal neuropiles called glomeruli, and gives rise to the lateral antennal lobe tract (l-ALT, new tract nomenclature after Ito et al., 2014) containing about 510 PNs. The dorsal hemilobe consists of about 77 glomeruli which send out about 410 PNs via the medial ALT (m-ALT) (Abel et al., 2001; Kirschner et al., 2006; Rybak, 2012) PNs from the two separate tracts respond to a similar stimulus space. For instance there is no apparent specialization for either floral odors or pheromones. Nevertheless, PNs of l-and m-ALT differ in physiological properties, like response latency, odor specificity and response dynamics (Müller et al., 2002; Krofczik et al., 2008; Nawrot, 2012; Brill et al., 2013; Carcaud et al., 2015), implying different functions. Both the anatomical layout and the physiological distinction make l-ALT and m-ALT candidates well suited for parallel processing of different stimulus aspects.

Having said that, the dual olfactory pathway also displays mirror-imaged like trajectories which could likewise implement coincidence detection. The m-ALT first innervates the MB and finalizes in the lateral horn (LH), known for innate odor responses (Gupta and Stopfer, 2012; Roussel et al., 2014; Strutz et al., 2014). The l-ALT runs exactly opposite, projects first to the LH and ends in the MB. This counter-rotating neuronal architecture thus produces a substantial temporal delay between the two tracts depending on which downstream neuron is activated in the MB. For instance, is a medial KC activated the l-ALT PNs will need a comparably longer time to reach that cell in contrast to the m-ALT. In comparison, is a more distal lateral KC activated the l-ALT will have reached the neuron earlier than the m-ALT (see Figure 4 in Rössler and Brill, 2013). This counter-rotating layout resembles detectors of coincidence for stimulus features such as delay lines known from the vertebrate auditory system, where sound localization is achieved by coincident input from neurons of both ears (Jeffress, 1948; Joris et al., 1998). A structural similarity that naturally has inspired speculations about a similar function in odor processing (Galizia and Rössler, 2010; Rössler and Brill, 2013).

Principle neurons of the MB, the Kenyon cells (KCs) receive highly convergent PN input (Caron et al., 2013; Gruntman and Turner, 2013). Moreover, PNs sent diverging output onto several KCs (Yasuyama et al., 2002; Leiss et al., 2009; Groh et al., 2012). Combined, both connectivity patterns lead to a temporally and spatially sparse KC population code (locust, e.g., Perez-Orive et al., 2002; honeybee, e.g., Szyszka et al., 2005; moth, e.g., Ito et al., 2008; fly, e.g., Turner et al., 2008). This code includes that individual KCs are activated only by highly coincident input from many PNs (Gruntman and Turner, 2013). Accordingly, synchronous activation of PNs has repeatedly been shown to be an important strategy for detection and learning of odors (e.g., Christensen et al., 2003; Martin et al., 2013; Riffell et al., 2013). Moreover, about 25% of PNs show odor specific latencies, which are shorter for l-ALT PNs than for m-ALT PNs (Krofczik et al., 2008; Brill et al., 2013). This latency code, in combination with the counter-rotating inputs, may lead to odor specific activation of different KC ensembles due to step-by-step coincidence between l-ALT and m-ALT PNs (Rössler and Brill, 2013).

While our knowledge about PN-tracts supports both, parallel processing and coincident delay lines, the two mechanisms put different constraints on the system. A prerequisite for an olfactory delay line would be that the information carried by the different tracts is combined. Accordingly, activity of individual neurons responding at the same time to the same stimulus should be highly coincident between the different tracts (see **Figure 4**, Rössler and Brill, 2013). And might also incorporate coincidental activity within tracts, which has been shown in other insects (Stopfer et al., 1997). Instead, parallel processing does not require the combination of inputs from different tracts, but might rather integrate information carried by neurons within the same tract.

To investigate how olfactory information is combined along the two tracts, we recorded odor responses of simultaneously active PNs and measured coincident activity within and between different PN subpopulations. Our results illustrate that coincidence is differently pronounced within each of the two tracts. Coincidence between tracts is present but does not outplay coincidence within the individual tracts. Taken together the findings strengthen the idea of parallel processing and the relevance of spike timing in coding of olfactory information in the olfactory system of the honeybee.

## Material and methods

### Animal preparation

Foragers of the European honeybee *Apis mellifera carnica* (Pollmann) were caught from a feeder filled with saccharose solution (Apiinvert, 50%) and harnessed and movement restrained according to standard routines. The brain was exposed by opening the head capsule. Glands, trachea and neurolemma were removed carefully. A reference electrode (chloride Ag-wire, 150 μm, AGT05100 WPI, Germany) was placed between the ocelli, a second electrode monitored proboscis movements by recording muscle activity. Tetrodes to record from the olfactory tracts were inserted outside the AL where l- and m-tract run in separate loops to the MB. Following electrode placement the brain was either covered with two component low viscosity silicone (Kwik-Sil, WPI, USA) or left untreated (Brill et al., 2014).

### Odor stimulation

Odor and control stimuli were delivered by a custom build olfactometer under constant stream of humidified and charcoal filtered clean air. Stimulation airstream was removed by an exhaust. The mean delay between stimulus expulsion from the olfactometer and the arrival at the animals antenna was 99 ms as estimated from Electro Antennogram (EAG, c.p below). Each animal was stimulated with the full set of 12 different odors in randomized order. The set comprised key elements of general plant odors (limonene, hexanal, 1-pentanol, 1-octanol, 2-octanone), natural plant odors (clove oil, orange oil, citral), and pheromones (geranylic acid, isoamylacetate, 1-hexanol, 2-heptanone) (Table 1). All stimuli were diluted 1:100 in mineral oil, applied in pulses of 500 ms and response measured repeatedly in 20 trials each. Mineral oil and pure air were applied as control stimuli.

**Table 1:** **Odor stimuli used in the experiments**.

**Odor**	**Abbreviation**	**CAS number**	**Odor type**	**Biological significance**
Spontaneous	spont			
Control air	ctr			
Citral	Cit	5392-40-5	terpen	floral and pheromone
Geranyl acid	Ger	459-80-3	terpen	floral and pheromone
Isoamylacetate	IAA	123-92-2	esther	pheromone
(+) Limonene	Lim	5989-27-5	terpen	floral
Clove oil	Clv	8000-34-8	natural blends	floral
Orange oil	Orng	8008-57-9	natural blends	floral
Hexanal	6-al	66-25-1	aldehyde	floral
1-Pentanol	1-5-ol	71-41-0	alcohol	floral
1-Hexanol	1-6-ol	111-27-3	alcohol	pheromone
1-Octanol	1-8-ol	111-87-5	alcohol	pheromone
2-Heptanone	2-7-ne	110-43-0	ketone	pheromone
2-Octanone	2-8-ne	111-13-7	ketone	floral

*Odor abbreviations, the chemical abstract service number (CAS), chemical group of odorants and their biological significance for the honeybee are given. The used trials per odor are 20, number of recorded bees is 12, and the number of single PNs from the l-ALT is 54 and m-ALT 65, respectively*.

For further details of stimulus application and data acquisition refer to Brill et al. (2013).

### Electrophysiology

#### Multi-unit recordings

Electrodes consisted of three micro-wires made of copper (polyurethane-coated, 15 μm diameter, Elektrisola, Germany) and glued together with melted dental wax (Brill et al., 2014). One of these electrode shanks was placed to record from the l-, a second from the m-ALT, both of which were connected to a switchable headstage (SH16, Tucker-Davis-Technologies, USA). A silver-wire reference electrode was placed between the ocelli. Signals were fed into a custom designed connection module (INT-03M, NPI, Germany) and transferred to a custom-made amplifier system consisting of 16 custom designed low noise differential amplifier modules (DPA-2FL, NPI, Germany). To control for a potential influence of muscle activity on multi-unit recordings, the activity of the proboscis muscle, M17 was monitored. Recordings from all channels were 5 k differentially amplified to the reference electrode, band-pass filtered (300–8000 Hz), and shank-wise differentiated, that is: potentials recorded from each micro-wire within one shank were pairwise subtracted from each other to eliminate interfering signals (e.g., muscle activity, electrical hum). Subsequently data was stored for offline analysis. Sampling rate was 31,250 Hz at 16 bit resolution on each channel.

#### Electro-antennogram (EAG) recordings

EAGs were measured from the antenna ipsilateral to multi-unit recordings in five bees tested with the complete odor panel. Low-resistance (< 0.5 M Ohm) borosilicate electrodes (1B100F-3,WPI,USA) were pulled with a horizontal filament puller (DMZ Universal Puller, Germany) and filled with 0.5 M KCL-solution. A tungsten electrode below the scapus of the same antenna served as reference. Signals were amplified first by an intracellular headstage (Gain 10, Model 1600, A-M-Systems, USA) and subsequently by the same custom build amplifier as the multi-unit recordings (Gain 100) and band-pass filtered (0.1–100 Hz). Prior to analysis recordings were smoothed offline with an algorithm provided by Spike2 (time constant 32 μs). Smoothed EAGs were averaged over repeated trials. Response onset was defined as the relative maximum preceding the steepest negative slope of the potential drop which demarcated an odor response (Meyer and Galizia, 2012).

#### Spike sorting

Spikes were sorted using established routines implemented in commercial software (Spike2, v7.4, Cambridge Electronic Design, England). Each channel was preprocessed by smoothing with a FIR-filter (time constant 80 μs) and DC removal (time constant 3.2 ms). Signals recorded from all three channels were used for spike- sorting, unless one of the channels had to be excluded due to insufficient signal-to-noise (SNR) ratio, in which case the remaining two channels were used. We performed semi-automated template-matched spike sorting with an amplitude threshold set to the mean spontaneous activity ±3 standard deviations. Spontaneous activity was recorded over at least 1 min of activity prior to odor-test trials. Templates were formatted in semi-automated fashion in time windows from −0.4 to 0.8 ms around each spike's peak. Units were clustered and sorted by applying the Spike2 built-in dialogs based on PCA and additional feature extraction. For more detailed description refer to previous publications based on the same dataset (Brill et al., 2013).

### Data analyses

After spike sorting individual units were judged with respect to responsiveness and reliability (see paragraph “identification of odor-response profiles in PNs” in Materials and Methods in Brill et al., 2013). We wanted to know if coincident activity is a mechanism that is potentially used by honey bee PNs to combine the odor information that is carried in their spike trains. To isolate odor related activity, we excluded trials without odor-responses as well as those that were corrupted by artifacts (e.g., hum from the mains, muscular activity etc.) We analyzed coincidence within each animal between simultaneously active PN units using cross correlation. From each of the 12 animals we extracted eight units on average. Altogether 102 units were included in the analyses. Based on electrode placement these units could be identified as either l-ALT (49 units, on average 4 per animal, minimum 2, maximum 6) or m-ALT PNs (53 units, on average 4 per animal, minimum 3, maximum 6) such that coincidence within and between tracts could be identified. Analysis routines were custom written in Matlab (2010a; The MathWorks, Inc.).

#### Detection of simultaneous odor responses across units

Our objective was to analyze simultaneous odor evoked activity in small ensembles of PNs. For this purpose we compared the activity between units within one animal and selected pairs that were responsive to the same stimulus. In brief, we detected for each individual unit which odors were effective in evoking responses. Subsequently we matched each unit's response spectrum to those of the other units in the same recording. This way we ended up with pairs of l-ALT, m-ALT (within tract) and l-m-ALT (between tracts) units that were simultaneously active.

In order to achieve the response detection for each unit, we employed a fully automated routine of five successive steps: (1) To detect responses from averaged trials we re-sampled to bins of 1 ms and averaged trials of repeated presentation of the same stimulus. (2) We estimated the rate function of this averaged trial by convolution with a symmetric smoothing filter (Savitzky and Golay, 1964, polynomial order 0, 301 ms width, Welch-windowed). (3) Baseline firing rate was estimated over an interval of 600 ms before stimulus onset. (4) A response was defined as a deviation from baseline ±2 standard deviations with duration of at least 50 ms in a time window from 0 to 600 ms post stimulus. Deviations above threshold correspond to excitations–deviations below baseline correspond to inhibitions. (5) If a response was indicated in the average trial, we repeated the procedure on the level of the underlying single trial spike trains. (6) If a response occurred in at least half of all single trials, we accepted the odor as a potent stimulus for the given unit. Setting the threshold for responsiveness to 25 or 75% did not significantly change the quantitative results (Brill et al., 2013). Trials without a response as well as inhibited responses (< 1%) were excluded from further analysis.

Control stimuli are expected to evoke no (air) or only weak (mineral oil) responses. In order to monitor baseline coincident activity we included all control trials into the analysis irrespective of whether or not a response was detected.

#### Cross correlation

We detected coinciding spikes between different units by estimating the cross-correlation function for simultaneously recorded spike trains carrying odor information.

After selecting those trials in which a given pair of neurons was active simultaneously, we estimated cross-correlation using the observed elapsed times from one spike in the first unit's train to all spikes in the second unit's train in time window υ. In repeating this procedure for every spike, we obtained for each unit pair the set of all possible differences between spike times for all simultaneous trains in the cross-correlation window −υ to +υ.

Next we estimated the density function of this cross-correlogram using a Gaussian kernel with a fixed bandwidth of 25 ms (σ = 5 ms). This procedure is equivalent to classical cross-correlation but avoids *a-priori* determination of fixed bin sizes with equal weight. The density function reflects the probability of simultaneous occurring events at a given time. It is normalized to the total number of events within the underlying data.

At our chosen bandwidth of 25 ms, 68% of all integrated events fall within the central 10 ms of the kernel. A timing that resemble the integration time at a possible post synapse of a KC receiving input from both of the correlated units (PNs) as was shown by modeling approaches in honeybee, locust, and moth (Perez-Orive et al., 2004; Cassenaer and Laurent, 2007; Finelli et al., 2008; Martin et al., 2013).

To account for stimulus induced and random coincidence of spiking events, we subtracted a shuffle predictor from the density function of the raw cross-correlogram. The shuffle predictor was obtained by the same routine as explained above but from non-simultaneous trains of the same neuron pair. Bootstrap resampling from this non-simultaneous cross-correlation yielded a 95% confidence interval. Coincident activity was accepted as significant when the density function of the raw cross-correlogram exceeded the upper bound of the 95% confidence interval of the shuffle predictor.

To quantify coincident activity within a pair of units, we calculated the *Coincidence Index (CI)*. *CI* is the summed Area from periods of significant coincident activity [*t*(D_95%_)] under the density function of the shuffle corrected cross correlogram (*D*_*cross*_ − *D*_*shuffle*_).

$$CI=((D_{cross}>D_{95\%})-D_{shuffle})$$

*CI* reflects the significant coincident activity exceeding the expected coincidence.

### Statistical testing

We hypothesized that coincident activity is differently distributed between tracts. To test this assumption we needed a non-parametric procedure suitable for samples of unequal size but dependent data. In using a bootstrap hypothesis test all these requirements were met. We proceeded as follows: Each time we tested two out of the three possible datasets (coincidence strength of l-tract, m-tract and lm-tract, respectively) against each other. From each set, we drew 500 bootstrap resamples, the same size as the smaller of the two underlying dataset. A bootstrap resample is defined as a random sample drawn with replacement from the empirical distribution. We calculated the population median for each of these resamples, which left us with two equal sized samples. Given that these two samples distribute around equal medians, subtracting one sample from the other should yield a distribution around zero. A hypothesis (H0) that can easily be tested by calculating the probability of the observed median and comparing it to a predefined level of significance (alpha). We set our alpha to 0.05 but corrected for multiple testing using the Bonferroni procedure, yielding a final alpha of 0.016, if all three possible combinations (l-tract:m-tract; l-tract:lm-tracst; m-tract:lm-tracts) were tested.

#### Correlation matrix

We wanted to test if strong odor responses go hand in hand with high coincident activity. For this purpose, we correlated the tuning to an odor with coincident activity. We extracted odor tuning as follows: We measured response magnitude of each of our 102 units to every of the 12 odors used for stimulation. Response strength was given by the peak rate of the evoked firing rate change. Next, we ranked response strength within each unit. We thus obtained for every odor 102 position ranks between 1 and 12. We extracted the matching coincidence activity as follows: For each unit we summed its strength of coincident activity with all other simultaneously recorded units that responded to a given odor. Like for the odor tuning, we ranked coincidence strength to each of the 12 stimuli within every unit. This left us with another vector of 102 position ranks for each odor. The relationship between these two population vectors describing odor response strength and coincident activity was assessed by correlation. High correlation is associated with similar ranks in both tuning vectors, low correlation with very different ranks.

## Results

When different neurons fire action potentials in close succession their activity is detected as coincident by a shared postsynaptic target. Coincident activity can be used by the neural system to combine information carried by individual neurons. We wanted to know whether this mechanism may be utilized by the medial and lateral AL tracts of the honeybees' dual olfactory pathway.

For this purpose we analyzed extracellularly recorded spike trains from a whole of 102 units (49 l-ALT, 53 m-ALT, 12 animals). Units from both tract of each animal were recorded simultaneously and stimulated repeatedly (20 trials each) with 12 different odors (Brill et al., 2013). Each unit responded simultaneously with at least one other unit of the same recording to at least one odor, resulting in a total of 397 combinations. Simultaneous odor responses occurred within one tract (85 unit pairs in l-ALT, 96 unit pairs in m-ALT) and between the two tracts (216 unit pairs l-ALT:m-ALT). Whenever a unit pair responded simultaneously to a set of stimuli it also displayed coincidental activity to at least one of these stimuli (100% congruence in l:l, 99,5% in l:m, 96% in m:m), however, not necessarily to every single of the effective stimuli. On average a given unit pair displayed coincident activity for 84% of the odors that were effective in driving simultaneous responses.

In order to remove spurious coincidence, we corrected for stimulus modulation of firing rates, by subtracting a shuffle predictor from the original cross correlogram (see methods). Further, we only considered coincident activity that exceeded a 95% confidence interval.

### Coincidence increases with odor stimulation

**Figure 2:**
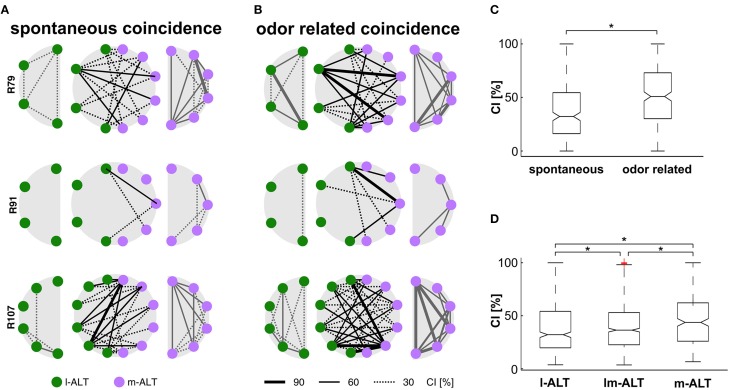
**Coincidental activity of single PNs within and between tracts**. **(A)** Significant coincident activity during recordings of spontaneous activity within l-ALT PNs (green, left row), m-ALT PNs (purple, right row) and between PNs of both tracts (middle row) from simultaneously multi-unit recordings in three honeybees as example. Lines indicate coincidence strength across PN pairs estimated by the coincidence index CI. **(B)** Significant coincident activity during odor stimulation trials. Colors and Indices as in **(A)**. **(C)** Quantitative measurement of coincident activity across all 392 combinations of recorded PN pairs, indicates significant increase of coincidence during odor stimulation (^*^Wilcoxon signed rank test, *p* < 0.001). **(D)** Coincident activity is highest within the m-ALT, followed by significant coincidence across PNs from both tracts. This qualitative observation is confirmed by a quantitative bootstrap hypothesis test (^*^Bonferroni correction, *p* < 0.002).

A prerequisite for every mechanism potentially encoding environmental information is that it should be more pronounced in the presence of a stimulus than in its absence. We compared recordings of spontaneous activity (Figure 2A) with odor stimulation trials (Figure 2B) to test whether this applies to coincidental activity of PNs within and between tracts. For this purpose we calculated a *Coincidence Index (CI). CI* reflects the significant coincident activity exceeding the expected coincidence.

Coincidence was present in both cases, but significantly higher under conditions of odor stimulation, than under spontaneous activity (Figure 2C; Wilcoxon signed rank test, *p* < 0.001). The amount of units expressing coincidental activity varied between recordings (animals). While coincidence was generally high in some ensembles (Figures 2A,B bottom row) it was rather low in others (Figures 2A,B middle row). Likewise, coincidence was not equally distributed between units of the same recording. While some units did not coincide with any other unit, others fired in close succession with many of the simultaneously recorded units, giving the impression of a “coincidence hub”. As a general rule, units with high spontaneous coincidence showed even stronger odor related coincidence. Considering our careful correction for spurious coincidence, this odor related effect cannot be attributed to the pure increase in firing rate that naturally follows excitatory responses.

### m-ALT units show more coincidence activity than l-ALT units

PNs from the l- and m-ALT differ both in morphology and their functional properties. In how far do they produce different degrees of coincidental activity?

As can be seen from visual inspection of ensemble plots alone, m-ALT units (Figure 2B right column) are more likely to produce coinciding spikes than l-ALT units (Figure 2B left column). Coincidence between tracts seems to appear more often than within the l-ALT but less pronounced as compared to activity within the m-ALT. This qualitative observation is confirmed by a quantitative bootstrap hypothesis test (Bonferroni correction, *p* < 0.002, Figure 2D): With a median *CI* of 44%, pairs of m-ALT units were significantly more prone to engage in synchronous firing than pairs of l-ALT units (*CI* = 32%) or mixed pairs of units from both tracts (*CI* = 36%). Strongest coincidence of unit-odor pairs occurred at relative times of 11 ms in the l-ALT, 10 ms in m-ALT and 9 ms between unit pairs of both tracts. A delay that is within the integration time of a postsynaptic KC as estimated by modeling approaches (Perez-Orive et al., 2004; Cassenaer and Laurent, 2007; Finelli et al., 2008; Martin et al., 2013). The unintended variability of electrode placement in the range of about 100 μm at the output of the AL is of minor relevance since a presumed neuronal conduction velocity of about 20 cm/s (Oleskevich et al., 1997) would add a temporal variance of less than 1 ms.

### Unit-pairs with similar odor-tuning do not synchronize stronger than controls

**Figure 3:**
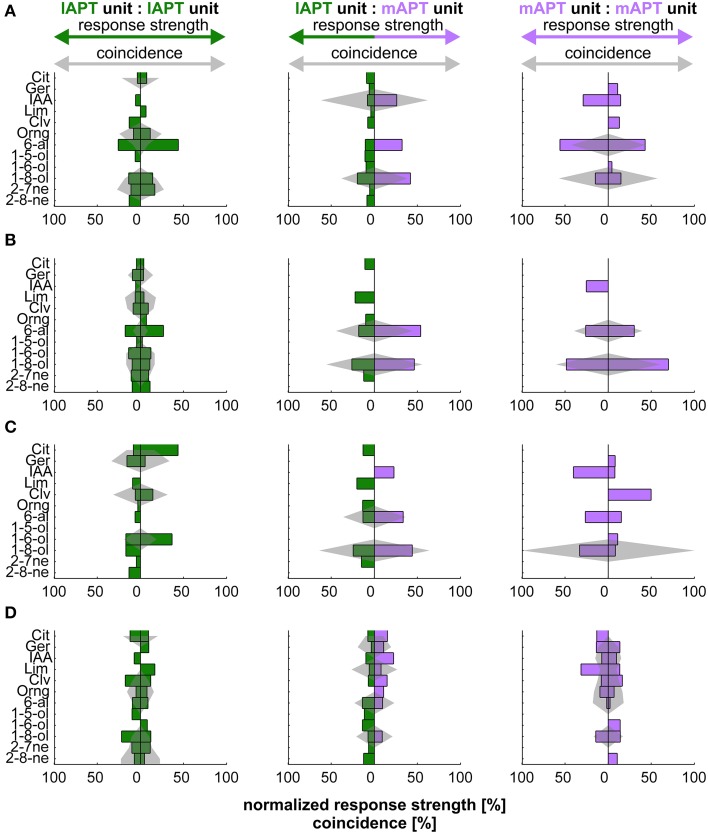
**Relationship between tuning and coincidence of exemplary unit pairs**. Odor tuning of individual units and coincidence of unit pairs within and between tracts. Barplots show tuning in percent response rate of the individual unit. Gray shaded areas indicate the strength of coincidence in percent of overall coincidence of a given pair. Rows **(A,B)** give examples of units with similar tuning (significantly positive correlated). Rows **(C,D)** show examples of unit pairs with dissimilar tuning (no correlation). Green: l-ALT; purple: m-ALT.

Neural codes typically involve the identity of individual neurons. Extracellular measurements sample randomly from groups of neurons with various identities, i.e., different odor specific characteristics. To access the possibility of an odor-specific code of coincidence that depends on unit identity, we investigated coincident activity of unit pairs with similar tuning properties. To assess similarity we ranked odor responses within each unit according to strength. We compared these tuning profiles of ranked odor responses by correlation. Positive correlation was indicative for similar tuning. Non-significant correlation around zero was indicative for dissimilar tuning. Altogether 50 unit pairs (l:l 8 pairs, m:m 20 pairs, l:m 22 pairs) showed significantly positive correlated tuning (Figures 3A,B). Compared to unit pairs with non-correlated tuning (344 pairs, random examples Figures 3C,D) similarly tuned units did not differ significantly in coincidence strength (bootstrap hypothesis test, *p* < 0.05). However, there was a tendency for similar tuned units being rather less well synchronized than others.

Qualitative assessment of this relation shows that this trend was particularly visible for unit-pairs within the l-ALT. Odors that evoked low response rates could produce strong coincidence (Figures 3A,C: left column, row **A**, orange Oil or row **C**, clove oil). Pairs, in which both l-ALT units showed a strong tuning to one particular odor, usually did not synchronize to that same odor (Figures 3A,C: left column, row A, hexanal or row **C**, hexanol). On the contrary, for unit pairs within the m-ALT at least one unit, less often both, showed prominent tuning to an odor if the pair produced notable coincidence (cp.: Figures 3A,B: right column, row **A**, hexanal, row **B**, octanol). For mixed pairs between the l-ALT and the m-ALT an odor that evoked strong rate responses in both units likewise exhibited strong coincidence (cp.: Figures 3A,C: middle column, row **A**, octanol, row **C**, octanol). Octanol and hexanal appeared to be particularly potent in driving both response rates and coincidence of m-ALT units.

### Odor identity is not reflected in a simple code of coincidence strength

Identification of biologically relevant odors is a key function of the olfactory system in behaving animals. Recent approaches have repeatedly described temporal relationships between neurons to be involved in this task (Stopfer et al., 1997; Perez-Orive et al., 2002; Riffell et al., 2013). We observed particularly strong coincidence amongst m-ALT units evoked by octanol and hexanal. Accordingly, we were curious whether odor identity was reflected by coincidental activity between units of the same or different tracts.

**Figure 4:**
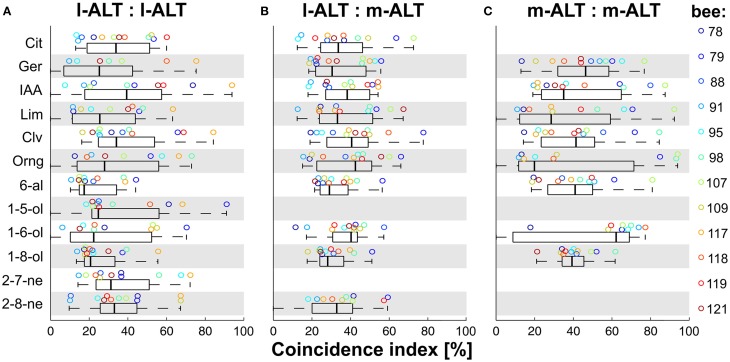
**Odor-specificity of coincident activity of PN pairs within and between tracts**. **(A)** Odor-wise analysis of coincidental activity of l-ALT PN pairs. **(B)** Odor-wise analysis of coincidental activity of mixed l-ALT to m-ALT PN pairs. **(C)** Odor-wise analysis of coincidental activity of m-ALT PN pairs. Note: since the m-ALT was shown to respond to odors sparsely with high odor specificity (lifetime sparseness), in 2 out of 12 odors not more than one PN fired in coincidence with l-ALT PNs, whereas in 2 out of 12 odors no m-ALT PN fired in significant coincidence with neither l- or m-ALT PNs. Box-plots of all analyzed PN pairs in response to the given odors with box-line as median, Box: first and third quartile, whiskers: first and ninth centile. Circles indicate the bee-wise median.

In a first step, we broke down the overall coincidence to the individual odor stimuli. For this purpose, we plotted the median *CI* distribution for each odor in the stimulus set, within and between tracts (Figure 4). Under the assumption that odor identity could be coded simply by the magnitude of coincidence, one would expect to see a systematic variation across animals in this distribution. Such a simple relationship however was not apparent. The median *CI* overlapped broadly between 0 and 80% for unit pairs within both tracts (Figures 4A,C). Between tracts, coincident activity was less dispersed but likewise overlapping (Figure 4B). None of the odors evoked a systematically high or low coincident activity. To the contrary, an odor that produced high *CI* scores in one recording could show low scores for another recording.

We conclude that a relationship between odor identity and coincident activity within an ensemble of units is not captured by a simple but inflexible code of coincidence strength.

### Odor tuning correlates with coincidence strength in m- but not in l-ALT units

**Figure 5:**
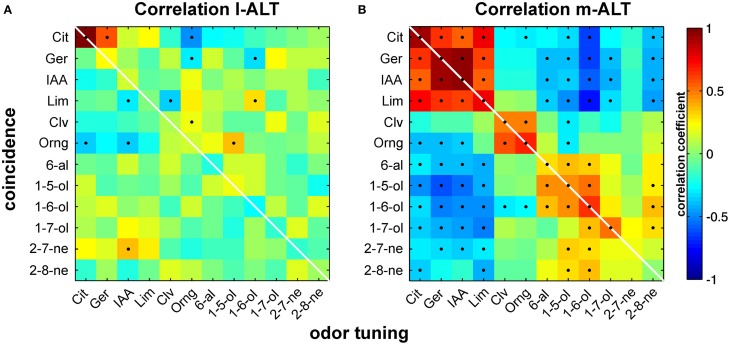
**Correlation of odor tuning with temporal coincidence of activity**. Correlation of odor tuning and spike time coincidences is high in PNs of the m-ALT and supports a potential relevance of spike timing in odor identity coding. Virtually no correlation between both parameters was found in the l-ALT. **(A)** Correlation of coincidence and odor tuning for l-ALT PNs. Odor tuning is given by ranked response strengths of individual PNs to a given odor. Coincidence is depicted by the ranked summed activity to a given odor, estimated by the coincidence index *CI*. Correlation for each odor is calculated across the entire PN population. Color heat map indicates the correlation coefficient. Significant correlations (*p* < 0.05) are indicated as black dots. **(B)** Correlation of odor tuning with coincidence strength in the medial antennal lobe tract (m-ALT).

We suspected a more flexible and thus more useful way of odor coding might get apparent when properties of individual units were taken into account. Based on the observations we made on single unit pairs within the m-ALT and between l- and m-ALT, we hypothesized that highly coincident activity would be more likely to appear for an odor that a given unit was better tuned to. To investigate the possibility of such a relationship we determined the response strength for every odor in each individual unit together with the strength of the corresponding coincidence. Next we correlated odor tuning with its corresponding coincident activity (Figure 5). The resulting correlation matrixes illustrate a marked difference between tracts: while the relationship appeared negligible within the l-ALT (Figure 5A), a strong pattern of significant correlation was apparent within the m-ALT (Figure 5B).

The emerging correlation matrix of the m-ALT met our expectations, since a positive correlation between odor tuning (e.g., to citral) and coincidence for the same odor (citral) was clearly visible. In practice: a unit that was strongly tuned to a given odor likewise showed strong coincidence, another unit with low tuning to the same odor would instead produce little coincidence. Surprisingly, positive correlation was not exclusively describing the relationship between tuning and coincidence to the same odor, but occurred similarly, even though generally less pronounced, to different odors belonging to similar chemical groups; e.g., citral and limonene (terpene); clove oil and orange oil (natural blends); pentanol and hexanol (alcohol). In contrast, negative correlations dominated the relationship between tuning and coincidence to odors from more distinct chemical classes; e.g., citral (terpene) and hexanol (alcohol). In fact, the characteristic pattern of positive and negative correlations might help the receiving mushroom-body circuits to discriminate and by this identify odors.

The marked lack of a similar relationship in the l-ALT is in congruence with both its previously documented rather stimulus unspecific responses (Brill et al., 2013) and its less pronounced coincident activity (c.f.: above). Our findings thus strengthen the notion of l-ALT and m-ALT being responsible for processing different stimulus properties and imply the utilization of different mechanisms for this purpose.

## Discussion

In the present work, we set out to investigate in what fashion olfactory information is combined along the separate tracts of the honeybee dual olfactory pathway. Does coincident activity between the tracts foster a detection of stimulus features comparable to the delay line system of the vertebrate auditory system? That is: do l-ALT and m-ALT PN show prominent coincident activity? Or is coincidence a potential mechanism to integrate information within the same tract, facilitating parallel processing of stimulus properties comparable to the prominently known parallel visual pathways? That is: do neurons within the same tract show prominent coincident activity? To answer these questions, we recorded odor responses of simultaneously active PNs and measured coincident activity within and between the different subpopulations. Our results illustrate that coincidence is differently pronounced within each of the two tracts. Coincidence between tracts is present but does not outplay coincidence within the individual tracts. Taken together the findings presented in this work support the notion of coincidence as an important mechanism in olfactory processing and, at the same time, strengthen the idea of parallel processing and delay-line coding in the dual olfactory system of the honeybee.

Synchrony within the AL has been shown to correlate with odor identity and intensity (Christensen et al., 2000; Lei et al., 2004; Riffell et al., 2009a,b) and has been suggested to represent a common encoding dimension for food odors and pheromones (Martin and Hildebrand, 2010). In agreement with these works, we find that PNs produce significant amounts of coincident spikes. More importantly however, this activity is specifically related to the presence of an odor stimulus. This relationship does not seem to be realized by the magnitude of coincidence alone. Accordingly, we could not find indications for a systematic relationship between coincidence strength and odor identity *per se*. Coincidence is the product of coordinated activity between at least two neurons. As such it represents the smallest unit of a processing network. Information processing in neuronal networks is believed to underlie higher order computations rather than an easy mathematical relationship (Laurent, 2002; Friedrich, 2013). Within the framework of network, our results and those of related works (Riffell et al., 2009a; Martin et al., 2013) suggest coincident activity to be a highly flexible mechanism that crucially depends on factors like the individual neurons' odor tuning and is as such suited to integrate biologically relevant information in upstream neurons.

In the same line of thinking, many studies have stressed the importance of coincidence detection by mushroom body KCs in the context of odor learning (Riemensperger et al., 2005; Cassenaer and Laurent, 2007; Gervasi et al., 2010) and odor discrimination (Perez-Orive et al., 2004; Jortner et al., 2007; Riffell et al., 2009a,b; Martin et al., 2013). Based on studies like these, the MB has been assumed as a coincidence detector for synchronous activity provided by the AL (Heisenberg, 2003; Rybak and Menzel, 2010; Davis, 2011). So far however, it has been difficult to disentangle what contribution is made by which type of neuron. Closing this knowledge gap is an important step in order to understand the function of different AL-neuron subpopulations (Galizia and Rössler, 2010). Moreover, it will help to develop refined models describing how upstream neurons in the MB make use of the information provided by the AL.

Using simultaneous dual-tract recordings we show for the first time coincidental activity that can directly be attributed to different morphological subclasses of AL PNs, which give rise to the lateral and medial tract projecting from the AL to the MB. The amount to which coincident activity is provided differs significantly within and between tracts. A finding that inspires to speculate about underlying mechanisms.

How is the striking difference in coincidence within l-ALT and m-ALT to be explained? What makes m-ALT PNs more likely to unite in synchronous firing than l-ALT PNs? And what impact might these differences have on upstream neurons? On the one hand, quantitative analysis of odor-evoked spike trains have attested higher overall firing rates in l-ALT units (Brill et al., 2013). On the other hand, qualitative observations of spiking patterns from both tracts have repeatedly led to descriptions of irregular and burst-like, phasic activity in m-ALT PNs contrasted by tonic activity in l-ALT PNs (Abel et al., 2001; Müller et al., 2002). When coding for comparable signals, bursts, in comparison with single spikes, have been shown to improve the SNR ratio (Sherman, 2001) and are suggested to improve information transfer between neurons (Lisman, 1997). Accordingly, the tendency of m-ALT PNs to display more burst-like activity might in fact outplay higher firing frequencies of l-ALT PNs when it comes to producing coincidence.

However, from the generally lower expression of synchronous firing in l-ALT PNs, it does not necessarily follow that coincidence within this tract is negligible. Even tough to a lesser degree than within the m-ALT, l-ALT units do produce significant amounts of coincident firing which upstream KCs could make use of. Input from PNs of the dual tracts might be processed differently: pyramidal neurons of the weakly electric fish have been shown to extract different aspects of stimulus information from coinciding burst-like and coinciding tonic spike trains (Oswald et al., 2004). If similar mechanisms exist in insect KCs has not yet been investigated.

Moreover, we should consider that KCs—just like vertebrate pyramidal neurons—might possess more than one type of coincidence detection (for reviews of coincidence detection in pyramidal neurons see Spruston, 2008). In our analysis of simultaneously recorded extracellular unit activity, we mimicked temporal summation by means of density estimation with a kernel about the length of possible postsynaptic integration. Our experimental approach did not allow to likewise consider coincidence detection as a result of spatial summation. Spatial summation crucially depends on large numbers of synaptic contacts. As a matter of fact, mature l-ALT PNs make more contacts with KCs than m-ALT PNs (Groh et al., 2012). Based on these morphological findings l-ALT PNs might thus be better suited to provide spatially coincident input, while m-ALT PNs give more temporally coinciding input, as indicated by our results.

In summary, the apparent differences of coincident activity as detected by our analysis illustrate that different mechanisms govern odor processing in each of the two tracts establishing the dual pathway. However, the final interpretation of these differences remains a matter of upstream KCs. In order to understand the interplay between PN output and KC response simultaneous recordings from all three types of neurons would be highly desirable.

Magnitude is not the only aspect in which coincident activity differs between l-ALT and m-ALT. We found a strong relationship of odor tuning vs. coincidence activity within the m-ALT, but not within the l-ALT. Based on these observations it is tempting to conclude that coincident activity of m-ALT PNs allows upstream KCs to specifically process odor identity; an assumption that is further supported by studies of stimulus specificity within the two tracts. Multi-unit recordings as well as calcium imaging from m-ALT PNs show significantly higher odor specificity than those of units from the l-ALT (Brill et al., 2013; Carcaud et al., 2015). A finding that could not be seen in a previous attempt using calcium imaging (Yamagata et al., 2009), most likely as a result of GABAergic mechanisms that impact PN activity in imaging approaches (Grünewald, 1999; Ganeshina and Menzel, 2001; Froese et al., 2014). Interestingly, recent imaging results from m-ALT glomeruli do show coding related to chemical groups of odors (Carcaud et al., 2012). A finding, that complements nicely with our tuning-coincidence correlation, where we likewise found similar relationships between odors of the same chemical group. In agreement with the higher odor specificity, m-ALT PNs seem to keep track of the single odorants if challenged by odor mixtures (Krofczik et al., 2008)—a strategy termed elementary odor coding. Joint activity of odor specific m-ALT PNs could allow for a combinatorial code of mixture embedded odor identity by the receiving KCs.

The picture that emerges from our results for l-ALT PNs is very different, particularly regarding odor identity coding. The striking lack of correlation between odor tuning and coincidence implies that joint activity of l-ALT PNs conveys poor information about odor identity. This however appears little surprising considering that l-ALT PNs are rather broadly tuned and express little odor specificity (Brill et al., 2013; Carcaud et al., 2015). In contrast to the m-ALT, l-ALT PNs are characterized by shorter latencies (Krofczik et al., 2008; Brill et al., 2013) and start to respond to odors already at very low concentrations (Yamagata et al., 2009; Schmuker et al., 2011; Carcaud et al., 2012). If challenged by odor mixtures they tend to respond to the mixture as a whole, rather than the single odorant (Krofczik et al., 2008). It might well be that any of these characteristics could produce significant correlation with coincident activity for l-ALT PNs but not m-ALT neurons - an assumption that due to the lack of suitable experimental data has to remain speculative for the time being.

Taken together, our results imply that coincidence within the tracts of the dual olfactory pathway serves different functions. These functions probably rely on the characteristics of the PN subgroups that allow for dedicated processing of different stimulus aspects. These findings support the suggestion that the dual olfactory pathway is ideally suited to implement parallel processing.

**Figure 6:**
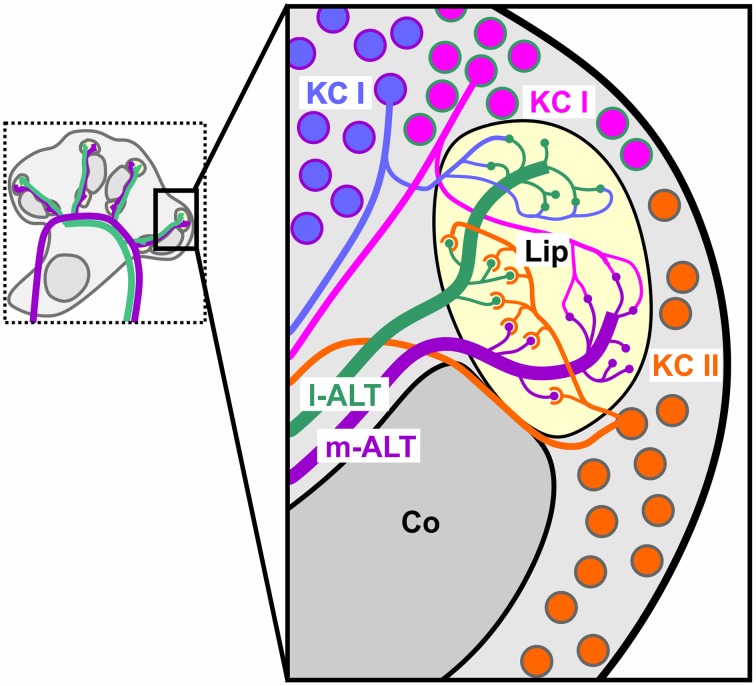
**Putative connectivity scheme of different KC subtypes and PNs**. Connections are partly inferred from morphological studies in other insect species. Non-compact KC (KC I, blue, magenta) most likely contact exclusively target regions of the m-ALT (purple) or l-ALT (green). Clawed KCs (KC II, orange) are likely to span over both the l- and m-ALT innervation areas within the olfactory lip.

Parallel processing keeps information from different sources separated. An olfactory delay line, in contrast, would combine information from different sources. As detailed above, the coincident activity we found within each tract gives strong support to the implementation of parallel processing by the dual olfactory pathway. However, we also found significant coincident activity across tracts. Even though joint activity between l-ALT and m-ALT PNs did not outplay activity within individual tracts, it produced significant amounts of coincidence which might just as likely be used by upstream KCs. Hence our finding complies likewise with the existence of olfactory delay lines. Could both of these mechanisms coexist? In fact, morphological evidence supports a possible implementation of both mechanisms in parallel (Figure 6). Mass-fill studies in different Hymenoptera have shown that PNs project to different sub-regions of the MB (Kirschner et al., 2006; Nishikawa et al., 2012). These separated inputs are received by various types of KCs. Some KCs make synaptic contacts only in one of the two PN input regions and likewise provide output to different regions (Strausfeld et al., 2000). That is, these types of KC maintain the possible separation of parallel pathways until its convergent input to extrinsic MB neurons (Rybak and Menzel, 1993). Another population of KCs, the so called clawed KCs (KC II; Mobbs, 1982), span their postsynapses across the innervation fields of both l- and m-ALT PNs (Strausfeld, 2002). Patch clamp recordings in the fly could show that these clawed KCs, on average, require coinciding input from about 4–6 PNs in order to be driven above threshold (Gruntman and Turner, 2013). Patch clamp experiments in cockroaches likewise support coincident activation of KCs, as indicated by their high action potential threshold (Demmer and Kloppenburg, 2009). Similarly, indications emanate from studies showing that input of PNs conveying information about different odors in changing temporal relationships evoke activity in KCs specifically tuned to certain asynchronous inputs (Saha et al., 2013). An observation that recently was also found in the vertebrate's olfactory cortex (Haddad et al., 2013). This subtype of KCs is hence predestinated to function as a coincidence detector for information coming from both tracts (Rössler and Brill, 2013).

In this perspective, subclasses of olfactory PNs of the honeybee first of all establish parallel pathways. Subclasses of KCs again could realize an implementation of both maintained parallel processing and delay-line like coincidence detection. Although our experimental paradigm favors the idea of parallel processing, further experiments which take KC activity directly into account need to prove, if delay line coding in the olfactory system does exist. Along this line further experiments also should test which of the mentioned coding strategies, either parallel processing or coincidence coding, benefit the animal in detecting complex odors.

As proposed earlier (Rössler and Brill, 2013) the dual olfactory pathway reminds of a delay-line system. Taking the proposed neuronal conduction velocity of about 20 cm/s in honeybees into account (Oleskevich et al., 1997), we assumed that indeed different delays between the PN tracts activate different KCs within the MB calyx at different places. In favor of a delay-line coding the measured maximal coincidence of about 10 ms within and 9 ms across tracts could add up on already measured latency differences between tracts and between individual PNs (Krofczik et al., 2008; Brill et al., 2013). The measured maximal coincidence as well as response latency would thus enable the system to implement an even more fine-scaled temporal and spatial KC activation pattern, a prerequisite for sparse coding.

While parallel processing is most probable important for tasks like odor identification and learning, an olfactory delay line and temporal coding could help e.g., to navigate along a concentration gradient to a food source or a mate. These abilities are obviously not only vitally important for honeybees and other Hymenoptera but likewise for behaviorally less complex insects like flies or moths. In recent years several attempts have been made to understand the possible functional relevance of the dual olfactory pathway of Hymenoptera (Abel et al., 2001; Müller et al., 2002; Krofczik et al., 2008; Yamagata et al., 2009; Brandstaetter and Kleineidam, 2011; Dacks and Nighorn, 2011; Rössler and Zube, 2011; Nishikawa et al., 2012; Brill et al., 2013; Carcaud et al., 2015). In the long run the knowledge gained from these studies might be transferred to insects with different tract layouts (Galizia and Rössler, 2010; Martin et al., 2011) and thus promote a more fundamental understanding of olfactory guided behavior.

### Conflict of interest statement

The authors declare that the research was conducted in the absence of any commercial or financial relationships that could be construed as a potential conflict of interest.
